# Household Socioeconomic Status and Antenatal Care Utilization Among Women in the Reproductive-Age

**DOI:** 10.3389/fpubh.2021.724337

**Published:** 2021-09-13

**Authors:** Yubing Sui, Rolle Remi Ahuru, Kaishan Huang, Muhammad Khalid Anser, Romanus Osabohien

**Affiliations:** ^1^School of Finance and Economics, Shenzhen Institute of Information Technology, Shenzhen, China; ^2^Department of Economics, Faculty of Social Sciences, University of Benin, Benin City, Nigeria; ^3^Center for Innovation and Entrepreneurship Education, Shenzhen University, Shenzhen, China; ^4^School of Public Administration, Xi'an University of Architecture and Technology, Xi'an, China; ^5^Department of Economics and Development Studies, Covenant University, Ota, Nigeria

**Keywords:** socioeconomic status, household, reproductive age, antenatal care, utilization

## Abstract

The study examined the effect of household socioeconomic status and other socio-demographic characteristics on antenatal care (ANC) utilization among 819 women within the reproductive ages across eight rural communities in Delta State, Southern part of Nigeria. Characteristics of the women were described using simple proportion and frequency. The chi-square test was used to examine factors that were significantly associated with a minimum of four (≥4) and eight (≥8) antenatal care contacts, which were respectively in line with the focused ANC and WHO's new guideline. The multivariable logistic regression was used to examine the determinants of a minimum of four and eight ANC. Statistical analyses were set at 5%. The results showed that 31.4% (257/819) and 2.2% (18/819) of mothers, respectively, made ≥ 4 and ≥ 8 ANC contacts in the course of their last pregnancies. According to the results, the odds for reporting 4≥ and ≥ 8 ANC improved with both wealth and educational attainment. Distance to the health center and cost are barriers to maternal care utilization and they reduce the odds for undertaking ≥ 4 and ≥8 ANC contacts. Women on higher media exposure were more likely to undertake ≥ 4 and ≥8 ANC contacts, and those on the highest media exposure were more likely to undertake ≥8 ANC contacts. Financing maternal care through health insurance and free maternal care significantly improves the odds to undertake ≥ 4 and ≥ 8 ANC contacts. Intervention programs should be designed to improve access to maternal care services and should expand education opportunities for mothers, improve household socioeconomic conditions, and encourage enrolment in health insurance and free maternal care in the study area.

## Introduction

Recent evidence shows that annually 295,000 women die from pregnancy-related complications and that sub-Saharan Africa (SSA) and South Asia bear the brunt of global maternal death (1, 2). In 2015, out of the 303,000 maternal deaths, 201,000 maternal deaths occurred among SSA countries (3). Maternal mortality ratio (MMR) in SSA is calculated at 351 per 100,000 live births, and SSA accounts for 66% of the global maternal-mortality burden (1). The life-time risk of pregnancy-related deaths is one in 37 among LDCs when compared to one in 7,800 for developed countries (1). Maternal mortality is a critical challenge in Nigeria. Annually, between 56,000 and 58,000 Nigerian women die from pregnancy-related complications. Recent evidence shows that the MMR in Nigeria at 917 per 100,000 live births (4).

Antenatal care (ANC) utilization has been reckoned as one of the means for reducing high maternal death in SSA (5, 6). Adequate and appropriate attendance at ANC provides the opportunity for timely detection of pregnancy-related risks and the introduction of appropriate measures to avert possible complications (7). It provides an opportunity for health tasks such as screening, health promotion, diagnosis, and disease prevention (8). ANC is a major healthcare tool that can help reduce the risks of still birth, preterm labor, and pregnancy-related complications (9). It is, therefore, imperative that women should receive adequate and quality ANC services to prevent unwanted complications and have a positive experience in the course of pregnancy (4). Analysis of a Nigerian case shows that ANC utilization rate is still at its suboptimal level. Recent estimates put ANC coverage in Nigeria at 57%. The ANC coverage of 57% falls short of the recommended 90% to achieve safe motherhood (10).

Several works have reported factors such as lack of money, poor quality care, transportation problem, the unfriendly attitude of healthcare providers, and disapproval of the spouse as factors that could hinder rural Nigerian women from utilizing evidenced-based cares (11). Poverty has been commonly reported as a barrier to maternal care utilization in Nigeria (11). In the light of this, the Federal Ministry of Health (FmOH) in 2007 recommended that all state governments in Nigeria should implement a policy of free maternal and childcare. By the end of 2010, over 50% of Nigerian states had implemented the policy (12). Also in recent years, there has been wide scale implementation of the policy of free maternal care at various levels of government in Nigeria (13).

The Delta State Government by the last quarter of 2007 implemented the policy of free maternal and childcare as a part of its health contributory scheme (14). The scheme covered free ANC services including drugs and ANC card, free vaginal and assisted vaginal delivery (forceps and vacuum), free post-abortion services, free management of ectopic pregnancy, free cesarean section (elective and emergency), and free laparotomy for obstetric complications (15). The aim of the scheme is to resolve financial barriers to maternal care utilization for all women in the state (16).

To reduce the number of preventable maternal deaths and to ensure that the Sustainable Development Goals target of reducing maternal mortality to <70 per 100,000 live births by the year 2030 is met, the WHO in 2016 came up with new guidelines for ANC (4, 7, 17). As part of the recommendations, the minimum number of ANC contacts should increase from four to eight or more. Evidence has shown that less frequent contacts were associated with maternal mortality (18). It is argued that increasing the minimum number of contacts from four to eight will reduce perinatal mortality by up to eight deaths per 1,000 live births (18). The new guideline stipulates that the first contact should be made in the first 12 weeks of gestation, with subsequent contacts at 20, 26, 30, 34, 38, and 40 weeks of gestation (4).

This study is undertaken to assess the influence of household socioeconomic status on the new antenatal care (ANC) guidelines of WHO among rural women in Delta State, Southern Nigeria. Household socioeconomic status is the main focus because we believe that in the era of free maternal care policy, the household socioeconomic status will have no significant impact on ANC utilization, since the free maternal care policy will resolve financial barriers and issues relating to the affordability of maternal care services (11, 19). The study focuses on rural communities in Nigeria due to the fact that maternal mortality is higher among rural Nigerian women, and evidence shows that several barriers to maternal care utilization confront rural Nigerian women (12, 16, 20). The study undertook the following research objectives: (i) It examined the effect of household socioeconomic status on a minimum of eight ANC contacts (ii) It examined the effect of other sociodemographic factors on a minimum of eight antenatal contacts (iii) It compared the determinants of the new recommended ≥ 8 ANC contacts and that of the focused ANC of ≥ 4.

## Materials and Methods

### Study Settings

The study area is Delta State, Southern Nigeria, while the study setting is Ughelli North local government area (LGA), which is one of the 25 LGAs that makeup Delta State. Ughelli North LGA lies between 9°45′N and 8°43′E with a total land mass of 818 square km. Administratively, the LGA comprises eleven wards with 105 communities embedded in the wards. Estimates from the 2006 population census put its population figure at 321,028, with a population density of 460.1 people per square km. Women account for approximately half of the population of people in the area. The main source of healthcare in the area is the primary healthcare centers (PHCs), although several private hospitals and one general hospital exist in the area and serve as first-level referrals for PHCs in the area. There are ~30 PHCs with a PHC density ratio of 18 PHCs per 10,000 of the population (21). Farming is the primary source of livelihood for people in the study area. Urhobo is the mother tongue of the dwellers in the communities. As it is a rural community, poverty and illiteracy rates are high (16). Though statistics on maternal mortality rate are not available, documented evidence shows that the rate of maternal care utilization is poor, and that several mothers still deliver outside health institutions (16).

### Study Design and Sampling Procedure

The study is a crosssectional household survey, and the population of the study comprises women aged (15–49) years who resided in the communities at the time of the survey. Adopting ANC utilization rate of 62.1% among rural women, which was reported by Okonofua et al. (12), an error margin of 5%; 1.96 critical value corresponding to a 95% confidence interval and a non-response rate of 10%; and the Cochrane' (22) sample size was used to obtain a minimum sample size of 819. The respondents for the study were selected using multistage sampling techniques. In stage 1, Delta State was randomly selected using simple balloting. In stage 2, one LGA was randomly selected out of the 25 LGAs. The selected LGA is Ughelli North, which has eleven administrative units. In stage 3, using simple balloting, four administrative wards were selected. In stage 4, simple balloting was used to select two communities from each of the administrative wards; hence eight rural communities were selected. In stage 5, household surveys were carried out in the eight randomly selected rural communities. All the houses in the communities were numbered and those households with at least one qualified woman were enlisted for the survey. The eligibility criteria include women within the reproductive ages (15–49) years; those who gave birth in the last 5 years preceding the survey; women who were married or were in consensual union, and those currently residing in the communities. Following the listing of households with eligible women, town criers were used to update the women on the visitation of the research team, to avoid them from either going to the farm or the market. On the day of the survey, eligible women were recruited for the survey. In households with more than one eligible woman, simple balloting was used to select one of them.

Non-proportional sampling method was used to apportion the women across the eight rural communities. Due to the lack of reliable data to work out proportional sampling, we assume that there were equal numbers of qualified women within the communities. The research initially proposed to survey 103 women from each of the community, but some communities had fewer women while others had more. More women were recruited from the communities with a larger number of qualified women to make up the observations of 819. The communities were consecutively surveyed until the required observations were obtained, and then the research team stopped.

### Research Instrument

The study used a pretested adapted questionnaire to elicit information from the women. The questionnaire was detailed and structured to capture the objectives of the study. It was organized into five sections, namely: household socioeconomic characteristics, husband characteristics, and reproductive characteristics of the woman; ANC, delivery care, and postnatal care experience; and barriers to maternal care utilization. It comprises mainly close-ended questions with options for respondents to choose from. The questionnaire was pretested by administering it to eight women in another community which shared similar socioeconomic characteristics with the research participatory communities. The scale reliability coefficient was reported as 0.70, which showed that the questions were internally consistent.

### Administration of Questionnaire

Trained field research assistants were used in collecting the data. Five research assistants, with B.Sc. degrees in health-related disciplines, were recruited for data collection. Research assistants were given a 2-day training on the study goals and objectives, study locations, study data collection, and ethics in the field survey. The questionnaire was administered through face-to-face interviews and was fielded in either Pidgin English or simple English language. An interpreter who understood Urhobo (the mother tongue of women in the research communities) was hired to enhance intelligibility between the research team and the women. During the fieldwork, research assistants were monitored by a field supervisor who performed spot checks and requested reinterview where necessary. The period of data collection lasted for 6 months (from June, 2018 to December, 2018).

### Outcome Indicators

Two outcome indicators were used in this study: (i) ≥ 4 antenatal contacts, where women who made at least four ANC contacts were coded 1; while those who made less than four or zero were coded 0. Visits ≥ 4 is the recommended number of contacts under the focused ANC care (23) (ii) ≥ 8 antenatal contacts where women who reported at least 8 ANC contacts were coded 1 while those who made less or zero were coded 0. Visits ≥ 8 is the recommended number of ANC visits by the new guidelines of WHO (18).

#### Independent Variables

Drawing from the models of maternal healthcare utilization and past studies on the utilization of facilities for maternal care, the following independent variables were included in the analyses: maternal age (16–19/20–24/25–29/30–34/35–39/40–49), age at marriage (14–17/18–24/25–29/≥30); maternal education (non-formal/primary/secondary/post-secondary); employment status of the woman (unemployed/employed); marital status (married/living together/divorced/separated/widowed); time involved in walking to the nearest health center (in minutes) (<30/30–59/≥60); cost of care as barriers to maternal care services (No/Yes); mode of healthcare payments (out-of-pocket/free maternal care/health insurance), and who pays for healthcare services (Respondents alone/husband alone/respondent and husband/others).

Exposure to the media index was generated by asking the participants questions on the number of times they watched television (TV) and listened to the radio. Three categories of responses were provided to participants. The response options were every day as opportunity allows, at least once a week, and not all. The option of every day as opportunity allows is scored 2 marks; at least once a week scored 1 mark, and 0 for the option of not at all. The responses were aggregated to generate five categories of exposure to media index: no exposure (0%), low-moderate (11–30%); moderate exposure (31–50%); high exposure (51–69%), and very high exposure (70–100%).

Household wealth quintile was constructed from data on household possession using the principal component analysis. This was based on questions on whether the household possessed some of the items such as radios, television, bicycles and facilities such as the type of floor, piped-borne water, electricity, refrigerator, cooking fuel, furniture, etc. The factor coefficient scores and *z*-scores were calculated. For each household, the indicator values were multiplied by the loadings and summed to produce household wealth index. Households were categorized into five groups: wealthiest, wealthier, average, poorer, and poorest (24, 25).

The selection of these variables was influenced by the healthcare utilization model advanced by Andersen and Newman (26). This is a behavioral model that explains the conditions that either promote or hinder the utilization of healthcare services (26). The model identified three factors or conditions that influence an individual to or not to use healthcare services. These factors are predisposing, enabling, and the need for care factors. Predisposing characteristic is a combination of demographic characteristics, health belief, and social structure. Demographic characteristics are the tendency of a woman to utilize maternal health services, and it covers maternal age, gender, family size, birth order (number of previous pregnancies), marital status, previous history of maternal complications, and general attitudes toward health services (26). Enabling characteristics explain the community and family resources at the disposal of the individual, which enables the individual to use healthcare services. Enabling characteristics include family resources (income, health insurance, access, availability of health facilities, and social network support) and community characteristics (the ratio of health personnel and facilities per 100,000 of the population cost of health services, and rural-urban location). Several studies have shown that accessibility is a core determinant of health-seeking behavior and health outcomes (4, 20). Need-based characteristics are the perception of the need for health services. Patients will always evaluate their need for medical help. According to Andersen and Newman (27), the need factor is the most critical determinant of health care utilization. In other words,'the perception of people for the need of health services and expected benefit from the use of services may motivate them to use the services. The analysis in this study draws extensively from the predisposing and enabling factors.

### Statistical Analysis

Data analyses were undertaken at three stages. In stage 1, frequency and simple proportion were used to describe the characteristics of the women. In stage 2, chi-square tests were used to explore the sociodemographic factors that were significantly associated with the two indicators at 10% significant level. At this stage, we cross tabulate the two indicators across the various sociodemographic factors. In stage 3, multivariable logistic regression was used to examine the determinants of ≥ 4 and ≥ 8 ANC contacts among the women. Statistical analyses were set at 5%. The 14th version of Stata was used for the analysis. The logit command was used to generate the adjusted odds ratio with 95% confidence interval.

### Ethical Consideration and Consent Approval

Approval to conduct the study was obtained from the Ethics Review Committee of the University of Benin Teaching Hospital with protocol number-ADM/E22/A/VOL.VII/14689. Permission to undertake the survey was sought from the leaders of the various communities (the Ovies and chiefs) where the survey was conducted. Also, consent was obtained from the heads of individual households from where participants were drawn. Finally, informed consent was obtained from the participants. In order to ensure anonymity, participants were identified with a unique identification number which was serially arranged in accordance with the communities from where the participants were drawn.

## Result

Table 1 represents the demographic characteristics of the women. A total of 819 women within the reproductive ages who gave birth in the last 5 years preceding the survey were recruited for the study, and their information was used for the analysis. Out of this, 31.1% were within the age group (35–39) years. The majority of the mothers got married within the age group (18–24) years. The highest proportion of the women reported non-formal education (31.0%). Roughly 95% of the respondents were employed, and details of employment showed that the majority of them were located in the informal sector (analysis withheld from the table). The majority of respondents were married and living with their spouses. Media exposure is moderate among women (46.4%). Majority of the respondents live within a distance of 60 mins walk to the nearest healthcare center. Majority of mothers fund maternal healthcare through free maternal healthcare services (84.4%). Considering household wealth quintile, 15.4% belonged to the poorer household wealth quintile, 31.5% on the average household wealth quintile, and 7.2% belonged to the wealthiest wealth quintile.

**Table 1:** Demographic characteristics of respondents (aged 15–49 years) covered in the household survey.

**Variables**	***N*** **(819)**	**%**
**Maternal age (in years)**		
15–19	20	2.4
20–24	69	8.4
25–29	115	14.0
30–34	154	18.8
35–39	254	31.1
40–44	200	24.4
45–49	7	0.85
**Age at marriage (in years)**		
14–17	81	9.9
18–24	539	65.8
25–29	133	16.2
≥30	66	8.1
**Maternal education**		
Non-formal	254	31.0
Primary	206	25.2
Secondary	230	28.1
Post-secondary	129	15.7
**Woman's employment status**		
Unemployed	38	4.6
Employed	781	95.4
**Marital status**		
Married	520	63.5
Living together	280	34.2
Divorced	9	1.1
Separated	6	0.7
Widowed	4	0.5
**Media exposure**		
Non-exposure	92	11.2
Low media exposure	180	21.9
Moderate exposure	380	46.4
High exposure	130	15.9
Very high media exposure	37	4.5
**Time involve in walking to the nearest health Centers (in minutes)**		
<30	226	27.6
30–59	240	29.3
≥ 60	353	43.1
**Cost of care as barriers to maternal care**		
No	463	56.7
Yes	356	43.5
**Knowledge of free maternal care services**		
No	126	15.4
Yes	693	84.6
**Mode of healthcare payments**		
Out-of-pocket	104	12.7
Free maternal care	691	84.4
Health insurance	24	2.9
**Who pays for health services^+^**		
Respondent alone	17	16.3
Husband alone	54	51.9
Respondent & husbands/others	33	31.7
**Household wealth index**		
**Poorest**	126	15.4
Poorer	210	25.6
Average	258	31.5
Wealthy	166	20.3
Wealthiest	59	7.2

+ *Deducted women who pay for maternal care through health insurance and free maternal care*.

### Bivariate Analysis

In this section, the Chi-square test was used to examine the variables that were significantly associated with a minimum of four and eight antenatal contacts (see Table 2). We cross-tabulated the two outcome indicators across the various sociodemographic characteristics. The test is conducted at a 10% significant level.

**Table 2:** Factors associated with antenatal care contacts among the women in the household survey.

**Variables**	**≥ 4**			**≥8**		
	**No (562)**	**Yes (257)**	**χ^2^/*p***	**No (801)**	**Yes (18)**	**χ^2^/*p***
**Maternal age (in years)**						
15–19	13 (65.0)	7 (35.0)	χ^2^ = 2.472	20 (100.0)	0 (0.0)	χ ^2^ = 3.906
20–24	50 (72.5)	19 (27.5)	*p* = 0.781	66 (95.7)	3 (4.3)	*p* = 0.563
25–29	76 (66.1)	39 (33.9)		111 (96.5)	4 (3.5)	
30–34	100 (64.9)	54 (35.1)		150 (97.4)	4 (2.6)	
35–39	179 (70.5)	75 (29.5)		250 (98.4)	4 (1.6)	
40–44	140 (70.0)	60 (30.0)		200 (100.0)	0 (0.0)	
45–49	4 (57.1)	3 (42.9)		4 (57.1)	3 (42.9)	
**Age at marriage (in years)**						
14–17	56 (69.1)	25 (30.9)	χ^2^ = 0.473	79 (97.5)	2 (2.5)	χ^2^ = 1.649
18–24	373 (69.2)	166 (30.8)	*p* = 0. 925	526 (97.6)	13 (2.4)	*p* = 0.648
25–29	88 (66.2)	45 (33.8)		132 (99.2)	1 (0.8)	
30–39	45 (68.2)	21 (31.8)		64 (97.0)	2 (3.0)	
**Maternal education**						
Non-formal	252 (99.2)	2 (0.8)	χ^2^ = 419.42	254 (100.0)	0 (0.0)	χ^2^ = 23.742
Primary	195 (94.6)	11 (5.4)	*p* < 0.001^*^	206 (100.0)	0 (0.0)	*p* = 0.023^*^
secondary	93 (40.4)	137 (59.6)		219 (95.2)	11 (4.8)	
Post-secondary	22 (17.1)	107 (82.9)		122 (94.6)	7 (5.4)	
**Woman's employment status**						
Unemployed	26 (68.1)	12 (31.9)	χ^2^ = 0.001	38 (100)	0 (0.0)	χ^2^ = 0.896
Employed	536 (68.6)	245 (31.4)	*p* = 0.978	763 (97.7)	18 (2.3)	*p* = 0.344
**Marital status**						
Married	356 (68.5)	164 (31.5)	χ^2^= 0.327	504 (96.9)	16 (3.1)	χ^2^ = 7.114
Living together	194 (69.3)	86 (30.7)	*p* = 0.849	279 (99.6)	1 (0.4)	*p* = 0.029^*^
Others	12 (63.2)	7 (36.8)		18 (94.7)	1 (5.3)	
**Media exposure**						
Non-exposure	91 (49.7)	92 (50.3)	χ^2^= 303.431	92 (100.0)	0 (0.0)	χ^2^ = 16.961
Low media exposure	156 (46.2)	180 (53.8)	*p* < 0.001^*^	175 (97.2)	5 (2.8)	*p* = 0.002^*^
Moderate exposure	290 (43.3)	380 (56.7)		375 (98.7)	5 (1.3)	
High exposure	24 (15.6)	130 (84.4)		126 (96.9)	4 (3.1)	
Very high media exposure	1 (2.6)	37 (97.4)		33 (89.2)	4 (10.8)	
**Time involve in walking to the nearest health Centers (in minutes)**						
<30	9 (3.9)	217 (96.1)		210 (92.9)	16 (7.1)	χ^2^ = 34.619
30–59	206 (85.8)	34 (14.2)	χ^2^ = 615.948	239 (99.6)	1 (0.4)	*p* < 0.001^*^
≥ 60	347 (98.3)	6 (1.7)	*p* < 0.001^*^	352 (99.7)	1 (0.3)	
**Cost of care as barriers to maternal care**						
No	259 (55.6)	207 (44.4)	χ^2^ = 86.608	445 (96.1)	18 (3.9)	χ^2^ = 3.346
Yes	303 (85.8)	50 (14.2)	*p* < 0.001^*^	356 (100.0)	0 (0.0)	*p* = 0.067^*^
**Knowledge of free maternal care services**						
No	115 (91.3)	11 (8.7)	χ^2^ = 35.476	126 (100.0)	0 (0.0)	χ^2^ = 14.033
Yes	447 (64.5)	246 (35.5)	*p* < 0.001^*^	675 (97.4)	18 (2.6)	*p* < 0.001^*^
**Mode of healthcare payments**						
Out- of- pocket	96 (92.3)	8 (7.7)	χ^2^ = 32.779	104 (100.0)	0 (0.0)	χ^2^ = 3.356
Health insurance	447 (64.7)	244 (35.3)	*p* <0.001^*^	673 (97.4)	18 (2.6)	*p* = 0.187
Free maternal care	19 (79.2)	5 (20.8)		22 (100.0)	0 (0.0)	
**Who pays for health services^+^**						
Respondent alone	15 (88.2)	2 (11.8)	χ^2^ = 1.362	17 (100.0)	0 (0.0)	χ^2^ = 0.576
Husband alone	52 (96.3)	2 (3.7)	*p* = 0.506	54 (100.0)	0 (0.0)	*P* = 0.750
Respondent & husbands/others	30 (90.9)	3 (9.1)		33 (100.0)	0 (0.0)	
**Household wealth index**						
**Poorest**	126 (100.0)	0 (0.0)	χ^2^ = 422.88	126 (100.0)	0 (0.0)	χ^2^ = 41.846
Poorer	209 (99.5)	1 (0.5)	*p* < 0.001^*^	210 (100.0)	0 (0.0)	*P* < 0.001^*^
Average	187 (72.5)	71 (27.5)		257 (99.6)	1 (0.4)	
Wealthy	31 (18.7)	135 (81.3)		154 (92.8)	12 (7.2)	
Wealthiest	9 (15.3)	50 (84.7)		54 (91.5)	5 (8.5)	

* *Variables that are significant at 10% significant level*.

+ *Deducted women who pay for maternal care through health insurance and free maternal care*.

### Factors Associated With a Minimum of Four Antenatal Contacts (≥ 4 Contacts)

The factors that are significantly associated with ≥ 4 antenatal contacts are maternal education, mass media exposure, time involved in walking to the nearest healthcare centers, and household wealth quintile (*P* < 0.10). The proportion of respondents who reported ≥ 4 antenatal contacts increases as educational attainment improves. Also, as the tertiles for media exposure improves, the proportion of the respondents who reported ≥ 4 antenatal contacts increases. As time involved in walking to the nearest health center increases the proportion of respondents who undertake ≥4 antenatal contacts reduces. A higher proportion of those who did not report healthcare costs as barriers to maternal care utilization undertook a minimum of four ANC contacts (44 vs. 14%). A higher proportion of those who had knowledge of free maternal care services undertook ≥4 ANC contacts (36 vs. 9%); whereas 20.8% of those who were under health insurance coverage undertook ≥ four ANC contacts; 35.3% of those who were taking advantage of free maternal care services did same, but only 7.7% of those who were funding maternal care out-of-pocket did the same. Finally, the proportion of respondents who reported ≥ 4 antenatal contacts increases as household wealth quintiles improves.

### Factors Associated With a Minimum of Eight Antenatal Contacts (≥ 8 Contacts)

The factors that are significantly associated with ≥ 8 antenatal contacts are maternal education, mass media exposure, time involved in walking to the nearest healthcare centers, household wealth quintile, costs of care as barriers to maternal care utilization, knowledge of free maternal care services, and marital status (*P* < 0.10). The proportion of respondents who reported ≥ 8 antenatal contacts increases as educational attainment improves. The highest proportions of mothers who undertake ≥ 8 antenatal contacts were those who reported very high media exposure. As the time involved in walking to the nearest health center increases the proportion of respondents who undertook ≥ 8 antenatal contacts reduces. A higher proportion of mothers who had knowledge of the free maternal care services undertook ≥ 8 ANC contacts compared with those who did not have such knowledge (2.6 vs. 0.0%). Although none of the women who reported costs as a barrier to maternal care undertook ≥ 8 ANC, 3.9% of those who did not report cost as a barrier undertook ≥ 8 antenatal contacts. Finally, the proportion of women who undertook ≥ 8 antenatal contacts increases as the wealth quintile improves.

### Multivariate Analysis

The multivariable logistic regression is presented in Table 3. The variables included were only those variables that were significant at 10% significant level using the Chi-square tests. The adjusted odds ratio, 95% confidence interval, and the probability values are presented.

**Table 3:** Logistic regression of determinants of antenatal care contacts among women in household survey.

	**≥ 4**		**≥8**	
**Variables**	**AOR (95% CI)**	***P***	**AOR (95% CI)**	***P***
**Maternal education**				
Non-formal education	1		1	
Primary	24.09 (0.85–0.98)	0.062^***^	21.42 (0.00–0.12)	0.064^***^
Secondary	673.57 (0.00–0.67)	<0.001^*^	437.08 (0.00–0.23)	<0.001^*^
Post-secondary	3140.47(0.00-0.31)	<0.001^*^	2797.62 (0.12–0.45)	<0.001^*^
**Media exposure**				
No exposure	1		1	
Low exposure	0.0 (0.0–0.00)	<0.001^*^	0.00 (0.00–0.09)	0.001^*^
Moderate exposure	0.0 (0.00–0.01)	<0.001^*^	0.01 (0.00–0.22)	0.004^*^
High exposure	1.73 (0.00–0.06)	<0.001^*^	0.14 (0.01–3.72)	0.236
Very high exposure	2.81 (0.00–0.07)	0.002^*^	1.72 (0.00–0.04)	0.001^*^
**Time involved in walking to the nearest health center (in minutes)**				
<30	1			
30–59	0.04 (0.0–0.02)	<0.001^*^	0.01 (0.00–0.02)	<0.001^*^
≥ 60	0.01 (0.0–0.0)	<0.001^*^	0.04 (0.00–0.00)	<0.001^*^
**Cost of care as barriers**				
No	1			
Yes	0.23 (0.02–0.78)	0.019^**^	0.13 (0.04–0.39)	<0.001^*^
**Knowledge of free maternal care services**				
No	1			
Yes	3.56e+7(na)	0.99	0.42 (0.07–2.65)	0.357
**Mode of healthcare payments**				
out -of- pocket	1			
free maternal care	7.52e+7(na)	0.01^**^		
Health insurance	1157.02	0.012^**^		
**Household wealth quintile**				
Poorest	1		1	
Poorer	0.45 (0.0–1.23)	0.345	1.23 (0.00–0.00)	<0.001^*^
Average	1.34 (0.01–0.40)	<0.001^*^	2.34 (0.00–0.23)	<0.001^*^
Wealthy	2.34 (0.01–0.78)	<0.001^*^	2.67 (0.00–0.45)	<0.001^*^
Wealthiest	2.89 (0.00–0.04)	<0.001^*^	3.78 (0.00–0.67)	<0.001^*^
**Marital status**				
Married			1	
living together			1.11 (0.43–2.86)	0.83
Others			0.02 (0.00–0.36)	0.008^*^

*AOR, adjusted odds ratio; COR, Crude odds ratio; CI, confidence interval; na, not available*.

*, **, *** *respectively represents odds that are statistically significant at 1%, 5%, and 10%*.

### Determinants of a Minimum of Four Antenatal Contacts (≥ 4 Contacts)

In Table 3, we present determinants of a minimum of four ANC contacts. The significant predictors of ≥ 4 ANC contacts were maternal education, mass media exposure, time involved in walking to the nearest health center, cost of maternal care as barriers to maternal care, mode of healthcare payments, and household wealth quintile. In reference to mothers who have non-formal education, those who reported primary (AOR=24.09; *p* = 0.062) secondary (AOR= 673.57; *p* < 0.001), and post-secondary education (AOR=3140.47, *p* < 0.001) were ~24, 674, and 3,141 times, respectively, as likely to undertake ≥4 ANC contacts. In reference to mothers who lived within a 30-min walk to the nearest health center, those who lived within (30–59) min (AOR = 0.04, *p* < 0.01) and ≥ 60 min walk (AOR = 0.01, *p* < 0.001) were significantly less likely to undertake ≥ 4 ANC contacts. In reference to mothers who have no media exposure, those who had high media exposure (AOR = 1.73, *p* < 0.001) and very high media exposure (AOR: 2.81; *p* = 0.02) were significantly more likely to undertake a minimum of four ANC contacts. Those who reported healthcare costs as barriers to maternal care utilization (AOR= 0.23, *p* = 0.019) were 77% significantly less likely to undertake ≥ 4 ANC contacts. In reference to mothers who pay for maternal care out-of -pockets, those who benefit from free maternal care (AOR = 7.52e+07, *p* = 0.001) and those were under health insurance coverage (AOR = 1157.02, *p* = 0.012) were significantly more likely to undertake ≥4 ANC contacts. In reference to women with the poorest quintile, those on the average (AOR = 1.34, *p* < 0.001); wealthy quintile (AOR = 2.34, *p* < 0.001), and the wealthiest quintile (AOR = 2.89, *p* < 0.001) were significantly more likely to undertake ≥ 4 ANC contacts.

### Determinants of a Minimum of Eight Antenatal Contacts (≥ 8 Contacts)

In Table 3, we present determinants of a minimum of eight ANC contacts. The significant predictors of ≥ 8 ANC contacts were maternal education, mass media exposure, cost of maternal care as barriers to maternal care, household wealth quintile, and marital status. In reference to mothers who have non-formal education, those who reported primary (AOR=21.42; *p* = 0.064), secondary (AOR= 437.08; *p* < 0.001), and post-secondary education (AOR: 2797.62, *p* < 0.001) were ~21, 437, and 2,798 times, respectively, as likely to undertake a minimum of eight ANC contacts. In reference to mothers who have no media exposure, those who have very high media exposure (AOR: 1.72; *p* = 0.01) were significantly more likely to undertake ≥8 ANC contacts. In reference to mothers who lived within a 30-min walk to the nearest health center, those who lived within (30–59) min (AOR= 0.01, *p* < 0.01) and ≥ 60 min walk (AOR = 0.004, *p* < 0.001) were significantly less likely to undertake ≥ 8 ANC contacts. Those who reported healthcare costs as barriers to maternal care utilization (AOR: 0.13, *p* < 0.001) were 87% significantly less likely to undertake ≥ 8 ANC contacts. In reference to women with the poorest wealth quintile, those on the poorer quintile (AOR=1.23, *p* < 0.001), average quintile (AOR= 2.34, *p* < 0.001), wealthy quintile (AOR= 2.67, *p* < 0.001), and wealthiest quintile (AOR= 3.78, *p* < 0.001) were significantly more likely to undertake ≥ 8 ANC contacts.

## Discussion of the Results

The study examined the impact of household socioeconomic status on a minimum of four and eight ANC contacts among 819 women who reported recent birth across eight rural communities in Delta State, Southern Nigeria. We found out that the coverage of ≥ 4 and ≥ 8 ANC contacts were respectively 31.4 and 2.2%. The prevalent rate of ≥ 8 ANC contacts is far below the rates reported by a Nigerian study (4, 7), a Ghanaian study (17), and a study in the Benin Republic (28). Intervention programmes should be implemented in the study area that will expand the coverage rate of ANC, particularly the newly recommended guidelines of WHO of eight contacts.

We noted that maternal education has significant positive impacts on both ≥ 4 ANC and the newly recommended ≥ 8 antenatal contacts. Our finding of a significant positive association between maternal education and ≥ 4 and the recommended ≥ 8 antenatal contacts conforms to past studies, both for Nigeria and elsewhere (7, 17, 20, 28–30). However, this finding contradicts the reports made by some other studies conducted in Nigeria and elsewhere (2, 31). The positive impact of education on ANC utilization is because educated women are more likely to be engaged in high-paying jobs and as such they can afford the costs of healthcare services. Also, educated women are aware of their basic human rights and may have gained higher health literacy. Consequently, they are more likely to overcome any form of barriers to maternal care utilization compared with their counterparts with lower education and lower health literacy, which are keys in determining the utilization of maternal care services (32).

We further observed that access to media showed increased odds for undertaking ≥ 4 and ≥ 8 ANC contacts, which conform to studies in Benin Republic (7), a Ghanaian study (17), an Ethiopian study (30, 33), India study (34), Bangladesh (35), and rural Malawi (36). The reason for this could be that frequently listening to the radio and watching television will increase the literacy of a person, which has been noted as a key determinant of maternal care utilization (17). According to Afu-Mensah et al. (37) information education through media influences the use of health services (17).

The result showed that time involved in traveling to the nearest healthcare center has a significant effect on ≥ 4 ANC and the newly recommended ≥ 8 antenatal contacts. In reference to those who lived within a 30-min walk to the nearest health center, those who lived within (30–59) min, and ≥ 60 min were less likely to undertake a minimum of four and eight ANC contacts. This implies that distance could serve as a barrier to maternal care utilization, particularly being able to meet the recommended number of contacts. This finding conforms to the reports made by past studies both for Nigeria and elsewhere (11, 14, 31). There will be a need to increase the coverage of PHCs in the study area.

We found out that women who reported costs of care as barriers to maternal care utilization were less likely to undertake ≥ 4 ANC and the newly recommended ≥ 8 antenatal contacts. This finding conforms to the reports made by past studies both for Nigeria and elsewhere (4, 12, 20, 32). Despite the free maternal care policy in the state, some women still experience costs as a barrier because of ignorance of the free maternal care policy, and due to the fact that free maternal care policy does not cover all costs, particularly indirect costs relating to transportation charges, waiting time in health facilities, opportunity costs of time in traveling to health centers, and the costs of drugs (11, 38).

Mode of payments has a significant impact on ≥ 4 contacts. For instance, mothers who pay through health insurance and those who utilize through free healthcare services were more likely to undertake ≥4 and ≥8 antenatal care contacts. This can be discussed within the framework of the conceptual model by Andersen and Newman (26), which advocated that medical insurance subscriptions are enabling factors in accessing health services. The finding is in conformity with studies for Nigeria and Ghana (39–42). According to Seidu et al. (32) health insurance and free maternal care enables women to afford healthcare and freely choose from various providers according to their needs without fear or worry about costs (32).

According to our research findings, we found out that compared with mothers from the poorest wealth quintile, those from the wealthy and wealthiest quintiles were more likely to undertake ≥ 4 and ≥ 8 ANC contacts. Similar findings have been reported in Nigeria (11, 29), Ghana (43), Tanzania (44), Uganda (45), Afghanistan (46), Ethiopia (47, 48), and Southern Mozambique (49). The reason for this may be that wealthy mothers will be able to afford the cost of healthcare services, which is a barrier to maternal care utilization in Nigeria (12).

### Strength and Limitations of the Study

The strength of the study is its community research design approach which enables 819 women within the reproductive age to be recruited to the study. Given the similarity of most rural communities in Nigeria, the findings of the study can be generalized to cover most of the rural Nigerian communities. Despite the utility of the findings of this study, it has noteworthy limitations. First, the data was obtained through verbal reporting and was not subjected to any form of validation such as the use of a health facility card. There is a possibility that respondents gave socially-desirable responses. Second, the limited scope of the study implies that the findings cannot be generalized to the entire Nigerian population. Third, the cross-sectional nature of the data could only give room for the association to be established, but no cause-effect relationship.

## Conclusion and Recommendations

This study examined the effects of household socioeconomic status on a minimum of four and eight antenatal contacts among 819 women who reported recent birth across eight rural communities in Ughelli North LGA in Delta State, Southern Nigeria. The results showed that the coverage rate of ≥ 4 and ≥ 8 ANC s among the women is low; hence there is a need for health intervention programmes in the study area to broaden coverage. The results showed that maternal education, media exposure, time involved in walking to the nearest healthcare centers, costs as barriers to maternal care, health insurance and free maternal care, and household wealth were the significant predictors of ANC utilization among the women. We recommend that educational opportunities should be expanded for women in the study area. At the primary and secondary education level, free tuition policy should be used to encourage female enrolment rate, and at tertiary level special cut off should be used to encourage female education. Multipronged approach should be used to improve household socioeconomic status. Such approaches should include vocational training programmes, income transfer policy, rural sector development scheme, and granting of free fertilizers and improved seedlings. Efforts should be made to create more awareness of the free maternal care policy in the study area. Community-based health insurance schemes should be expanded in the study area. Efforts should be made to improve the coverage of PHCs in the study area. To this end, one PHC should be sited within every 2 km radius in the study area. In addition to absolving pregnant women of ANC charges, other support should be provided to pregnant women which include subsidization of transportation costs, free drugs, and family outreach services. Furthermore, more communication using the vehicles of mass media to enlighten women in the study area on the importance of the new ANC guidelines by WHO will be helpful in facilitating behavioral changes.

## Data Availability Statement

The original contributions presented in the study are included in the article/supplementary material, further inquiries can be directed to the corresponding authors.

## Ethics Statement

Approval to conduct the study was obtained from the Ethics Review Committee of the University of Benin Teaching Hospital. Permission to undertake the Survey was sought from the leaders of the various communities where the Survey was conducted. Also, consent was obtained from the Heads of individual households where participants were drawn from. Finally, informed consent was obtained from participants. Participants were informed of the purpose of the study and were assured of confidentiality. They were educated that they had the choice to discontinue the study if they had wish without consequences. Finally, participants were made to sign a consent form which showed that they understood very well what was explained to them and that they also give their consents to partake in the Survey. No names or specific contact information was obtained from participants as they were identified by unique numbers.

## Author Contributions

RA conceived the study. YS designed the study and drafted the manuscript. KH and RO supervised the study. RA supervised data collection, entry and cleansing. RO and KH prepared the first draft. All authors reviewed the literature, read and approved the manuscript and agreed to be accountable for the final manuscript. RO uploaded the manuscript in the journal's website and did the follow up.

## Funding

This work is supported by the Humanities and Social Science Research Projects of Ministry of Education of the People's Republic of China (Funding No.18YJC790142). In addition, the study received funding from Research Center of Scientific Finance and Entrepreneurial Finance of Ministry of Education of Sichuan Province (Funding No. KJJR2021-013).

## Conflict of Interest

The authors declare that the research was conducted in the absence of any commercial or financial relationships that could be construed as a potential conflict of interest.

## Publisher's Note

All claims expressed in this article are solely those of the authors and do not necessarily represent those of their affiliated organizations, or those of the publisher, the editors and the reviewers. Any product that may be evaluated in this article, or claim that may be made by its manufacturer, is not guaranteed or endorsed by the publisher.
